# Forecasting influenza A pandemic outbreak using protein dynamical network biomarkers

**DOI:** 10.1186/s12918-017-0460-y

**Published:** 2017-09-21

**Authors:** Jie Gao, Kang Wang, Tao Ding, Shanshan Zhu

**Affiliations:** 1 School of Science, Jiangnan University, Wuxi, 214122 China; 2 Key laboratory of Systems Biology, Shanghai Institutes for Biological Sciences, Chinese Academy of Sciences, Shanghai, 200031 China

**Keywords:** Influenza A pandemic, Protein dynamical network biomarker (PDNB), The critical state, The outbreak state

## Abstract

**Background:**

Influenza A virus is prone to mutation and susceptible to human beings and spread in the crowds when affected by the external environment or other factors. It is very necessary to forecast influenza A pandemic outbreak.

**Methods:**

This paper studies the different states of influenza A in the method of dynamical network biomarkers. Through establishing protein dynamical network biomarkers of influenza A virus protein, a composite index is ultimately obtained to forecast influenza A pandemic outbreak.

**Results:**

The composite index varies along with the state of pandemic influenza virus from a relatively steady state to critical state before outbreak and then to the outbreak state. When the composite index continuous decreases for 2 years and increases of more than o.1 suddenly, it means the next year is normally in the outbreak state. Therefore, we can predict and identify whether a certain year is in the critical state before influenza A outbreak or outbreak state by observing the variation of index value. Meanwhile, through data analysis for different countries influenza A pandemic outbreak in different countries can also be forecasted.

**Conclusions:**

This indicates the composite index can provide significant warning information to detect the stage of influenza A, which will be significantly meaningful for the warning and prevention of influenza A pandemic.

## Background

It is proved that there is a kind of common critical phenomenon in lots of complex biological process, i.e. a relative stable state quickly enters into another state after a critical point in a very short period of time [1, 2]. There is the kind of critical phenomenon for influenza A, because it needs only a very short period of time quickly from a relative stable state to outbreak state after a critical point. Thus in order to timely and effectively prevent and control the outbreak of influenza A pandemic, the key lies in predicting the critical point before the outbreak.

At present, influenza A is studied from all aspects. Pan et al. found that the spatio-temporal network that connects the cities with human cases along the order of outbreak timing emerges two-section-power-law edge-length distribution, using the empirical analysis and modeling studies [3]. Chang et al. studied the vaccine for influenza, so as to achieve the effect of prevention of influenza [4]. Banerjee et al. made full comparisons for the structural features of all H1N1 HA gene sequences and the composition of global amino acid to make it possible to depict the developing trend of influenza A [5]. He et al. also made in-depth studies to identify HA protein epitopes of avian influenza virus [6].

This paper studies the different states of influenza A using DNB. Through establishing PDNB of influenza A virus protein and using the nature of DNB, a composite index is ultimately obtained to forecast influenza A pandemic outbreak. The composite index varies along with the state of pandemic influenza virus from a relatively steady state to critical state before outbreak and then to the outbreak state. Therefore, we can predict and identify whether a certain year is in the critical state before influenza A outbreak or outbreak state by observing the variation of index value. This indicates the composite index can provide significant warning information to detect the stage of influenza A, which will be significantly meaningful for the warning and prevention of influenza A pandemic. Meanwhile, through data analysis for different countries influenza A pandemic outbreak in different countries can also be forecasted.

## Methods

### DNB analysis

The concept of network biomarkers is set up with the development of high-throughput genomic technologies and the systematic and multidimensional study of molecular expression profiling [7, 8]. This concept refers to a series of markers as well as their mutual relations and has been proposed as a new marker type [9]. Compared with traditional biomarkers, these markers can accurately distinguish disease states for taking the links between the molecules into consideration [10, 11]. However, it is used to diagnose the states of diseases, not for the detecting the critical point before the outbreak of diseases.

The method of dynamic network biomarkers focuses on the detection and assessment of different stages of the disease in the development of disease and shows it is a time-dependent method [12]. It studies the location changes of the markers over time and the relationship among network markers over time changing and then constructs three-dimensional images showing the interaction relationship between the markers. Therefore the study of Network markers focuses on the molecular interactions and distinguishes normal and disease states, and the study of dynamic network markers focuses on dynamic changes, which is helpful to discover the marker accurately and comprehensively and further to distinguish the state of disease before outbreak. It not only does not depend on the method of small sample excavation mode markers, but also make it easier for clinical application. At the same time it can be used in future studies to find early warning signals in any biological process, such as differentiation, senescence and cell cycle of each phase as well as key change.

### Defining PDNB

**Fig. 1:**
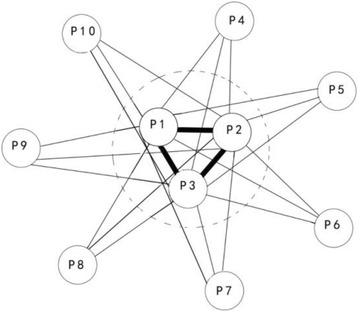
Protein dynamical network biomarkers. The protein that values of *CV*_*s*_ are the top three are defined as core protein (*CP*), and the others are no-core protein (*NP*). *CP* is a set of high confidence interactions of proteins, which forms a sub-network called the protein dynamical network biomarkers

Firstly, taking hemagglutinin (HA) protein as an example, we suppose that a HA protein marked y is linked sequentially by t numbers of amino acids. Its amino acid sequence is represented as *y* = *x*_1_ *x*_2_ ⋯ *x*_*t*_, in which *x*_*i*_∈ {A, V, L, I, P, F, W, M, D, E, G, S, T, C, Y, N, Q, K, R, H}; *i* = 1 , 2 ,  ⋯  , *t*. We suppose s-1-th year have m numbers of influenza virus HA proteins all over the world and its amino acid sequence is represented as *y*_*s* − 1 , 1_ , *y*_*s* − 1 , 2_ ,  ⋯  , *y*_*s* − 1 , *m*_. Meanwhile, We suppose s-th year have n numbers of influenza virus HA proteins all over the world and its amino acid sequence is represented as *y*_*s* , 1_ , *y*_*s* , 2_ ,  ⋯  , *y*_*s* , *n*_. The amino acid number of the *y*_*i* , *j*_ is marked *c*_*i* , *j*_,where *i*=s-1,s; *j* = 1 , 2 ,  ⋯  , *q*;*q* = max {*m*, *n*}. Sequentially selecting the i-th amino acid for *y*_*s* − 1 , 1_ , *y*_*s* − 1 , 2_ ,  ⋯  , *y*_*s* − 1 , *m*_ to form a new amino acid sequence is defined as *Z*_*s* − 1 , *i*_, and then take out the one of the largest number of amino acids. If the maximum number of amino acids has two or more than two, we take the first amino acid without loss of generality. At the same time, it is marked *x*_*i*_, where *i* = 1 , 2 ,  ⋯  , *k*;*k* = max {*c*_*s* − 1 , 1_, *c*_*s* − 1 , 2_,  ⋯ , *c*_*s* − 1 , *m*_}.We individually connect them in order to form a new amino acid sequences (*U*_*S* − 1_ = *x*_1_ *x*_2_ ⋯ *x*_*k*_) and then separately compare with corresponding amino acids of *y*_*s* , 1_ , *y*_*s* , 2_ ,  ⋯  , *y*_*s* , *n*_ one by one. If they are different, the assignment is 1, on the contrary the assignment is 0. Therefore, n new sequences are represented by *E*_*s* , 1_ , *E*_*s* , 2_ ,  ⋯  , *E*_*s* , *n*_ are obtained in s-th year. Then we calculate their mean (M), standard deviation (SD) and coefficient of variation (CV). Their computation formulas are as follows:

1 $$ {M}_s=\frac{\sum_{i=1}^nf\left(s,i\right)}{n} $$

2 $$ {SD}_s=\sqrt{\frac{\sum_{i=1}^{\mathrm{n}}{\left(f\left(s,i\right)-{M}_s\right)}^2}{n}} $$

3 $$ {CV}_s=\frac{SD_S}{M_s} $$

where *f*(*s*, *i*) represents the frequency of occurrence of one in sequence *E*_*s* , *i*_. Similarly, we calculate M, SD and CV of the other nine proteins. The protein that the top three values of *CV*_*s*_ are defined as core protein (*CP*), and the others are no-core protein (*NP*). *CP* is a set of high confident interactions of proteins, which forms a sub-network called influenza A virus proteins of protein dynamical network biomarkers (see Fig. 1).

### Defining forecasting index

The frequencies of the 20 kinds of amino acids can be calculated through the computation formulas as follows:

4 $$ {f}_{x_i}(s)=\frac{\sum_{j=1}^n{f}_{x_i}\left(s,j\right)}{n} $$

where ${f}_{x_i}\left(s,j\right)$ represents the frequency of occurrence of amino acid *x*_*i*_ in amino acid sequence *y*_*s,j*_. Therefore, we can get a 23 dimensional characteristic value vector of HA protein. By the same way, the ${f}_{x_i}(s)$ of the other nine proteins can be calculated in turn, so we can get a characteristic value matrix (X=[*V*_1_(*s*), *V*_2_(*s*),  ⋯ , *V*_10_(*s*)]), where *V*_*t*_(*s*) represents the characteristic value vector of the t-th influenza A protein, *t* = 1 , 2 ,  ⋯  , 10. Defining the characteristic distance between proteins:

5 $$ {d}_{vw}=\sqrt{{\left({M}_{vs}-{M}_{ws}\right)}^2+{\left({\sigma}_{vs}-{\sigma}_{ws}\right)}^2+{\left({CV}_{vs}-{CV}_{ws}\right)}^2+\sum_{i=1}^{20}{\left({f}_{vx_i}(s)-{f}_{wx_i}(s)\right)}^2} $$

where *v* and *w* respectively represents the *v*-th and the *w*-th protein.

The core proteins are not only the universal indicators to detect the complex outbreak signal of influenza A, but also the dominant or driving network of the whole protein system in the development, mutation and outbreak of the critical stages. In fact, the dominant network breaks through the limits of variation in the first time, first enters to the state of variation, and then affects other proteins and lead to the transfer of the entire system. Therefore, the determination of the dominant network can not only detect system in the critical state before break out, also help to reveal the underlying mechanism of influenza A virus proteins from the dimension of dynamic network. By combining the above properties of the core proteins, we can get a composite index:

6 $$ I=\frac{\overline{CV_k}\cdotp \overline{CP_{cd}}}{\overline{NP_{\mathrm{c}d}}} $$

Where $\overline{CV_k}$ represents the average value of the core proteins’ *CV*_*s*_, $\overline{CP_{cd}}$ is the average value of the characteristic distance between the core proteins, $\overline{NP_{cd}}$ is the average value of the characteristic distance between the core and non-core proteins.

When I_s-3_ > I_s-2_ > I_s-1_ and I_s_-I_s-1_ > 0.1, it can be concluded that *s* + 1 year is in the outbreak state.

Although the amino acid sequence of each protein will fluctuate randomly, the composite index can provide significant early warning information when the influenza A virus is close to the critical state before the outbreak or the outbreak state.

## Results

### Forecasting influenza A pandemic outbreak

Ten of proteins for influenza A virus are hemagglutinin (HA), matrix protein, matrix protein 2, neuraminidase, non-structural protein 1, non-structural protein 2, nucleocapsid protein, PA RNA polymerase, PB1 RNA polymerase and PB2 RNA polymerase. They are composed of 20 different amino acids link to form polymers. This paper selects influenza A virus protein sequences from 1934 to September 2016 from the NCBI website (http://www.ncbi.nlm.nih.gov/genomes/FLU/Database/nph-select.cgi?go=database), lots of data before 1934 are absent.

As shown in Table 1, by using the above methods to calculate the composite index of the 1934 to September 2016. However, we can’t figure out the composite index of some years, because some data in 1937–1942, 1944–1945, 1952–1956 years are absent.

**Table 1:** Composite index values from 1934 ~ 2016

Year	I	Year	I	Year	I
1934	0.723227	1971	0.728516	1995	0.164168
1935	0.962083	1972	2.379322	1996	0.611397
1936	0.43403	1973	0.79888	1997	0.711091
1943	0.543059	1974	0.527835	1998	0.629408
1946	0.441866	1975	0.801294	1999	0.781102
1947	0.851604	1976	2.275519	2000	0.710281
1948	0.448092	1977	2.182157	2001	0.295353
1949	0.760293	1978	0.438169	2002	0.660193
1950	0.734349	1979	0.32697	2003	0.465805
1951	1.027341	1980	0.746082	2004	0.45421
1957	0.859728	1981	0.455632	2005	0.772595
1958	0.949281	1982	0.650454	2006	1.595902
1959	0.474866	1983	0.449789	2007	0.476057
1960	0.550811	1984	0.354632	2008	0.798138
1961	0.708772	1985	0.939702	2009	1.344778
1962	0.08078	1986	1.166947	2010	0.962241
1963	0.980012	1987	0.655971	2011	0.740067
1964	0.650854	1988	1.033681	2012	0.635735
1965	0.527201	1989	0.912527	2013	0.606009
1966	0.500452	1990	1.898656	2014	0.806321
1967	0.666783	1991	1.248818	2015	2.516147
1968	2.31271	1992	1.032401	2016	0.843627
1969	1.081257	1993	1.187957		
1970	0.405805	1994	1.225976		

### Forecasting influenza A pandemic outbreak in pandemic occurrence place

Through influenza A virus protein data analysis for different countries influenza A pandemic outbreak in different countries can also be forecasted. Take China as an example, this paper selects influenza A virus protein sequences occurred in China from the NCBI website to forecast influenza A pandemic outbreak in China. Whereas lots of data in 1954–1956, 1958–1963, 1965, 1967 years are absent, all data before 1954 and in 2016 year are absent.

As shown in Table 2, by using the above methods to calculate the composite index of the 1954 to 2015. However, we can’t figure out the composite index of some years, because lots of data in 1954–1956, 1958–1963, 1965, 1967 years are absent, all data before 1954 and in 2016 year are absent.

**Table 2:** Composite index values in China from 1957 ~ 2015

Year	I	Year	I	Year	I
1957	0.835938	1982	0.566947	1999	0.702834
1964	0.464712	1983	0.489632	2000	0.718456
1966	0.571425	1984	0.467346	2001	0.645986
1968	2.198217	1985	0.896537	2002	0.673956
1969	1.217563	1986	1.025471	2003	0.653958
1970	0.645643	1987	0.637485	2004	0.543824
1971	0.756451	1988	0.653873	2005	0.854835
1972	2.189534	1989	0.712854	2006	1.632657
1973	0.815746	1990	0.762368	2007	0.549367
1974	0.776357	1991	0.772543	2008	0.924375
1975	0.780346	1992	0.805734	2009	2.184658
1976	0.855628	1993	0.784756	2010	0.843569
1977	0.902765	1994	0.812367	2011	0.483967
1978	0.536847	1995	0765347	2012	0.513975
1979	0.422576	1996	0.714358	2013	0.563954
1980	0.468257	1997	0.726856	2014	0.606837
1981	0.496629	1998	0.683546	2015	0.663854

## Discussion

### Forecasting influenza A pandemic outbreak

The dynamic network markers of Pandemic influenza virus vary in the whole process from a relatively stable state to the critical state before outbreak as well as the outbreak state, which results in the status transfer of the entire network and finally results in fluctuations in the composite index. Therefore, by observing the transformation of the composite index, we can predict the critical state before the outbreak of pandemic influenza and the outbreak state.

**Fig. 2:**
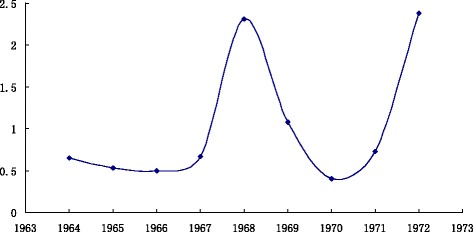
*Trend Chart* of composite index values from 1964 ~ 1972. *Horizontal axis* represents the year from 1964 ~ 1972, *vertical axis* represents the composite index value

The flu broke out in Hong Kong in 1968 and continued until 1969, of which 7.5 million people died. In 1972, influenza broke out in Henan Province and quickly spread to the entire province. As shown in Fig. 2, in 1964, the composite index value is 0.650854, 1965 is 0.527201; 1966 is 0.500452; 1967 is 0.666783; 1968 is 2.31271; 1969 is 1.081257; 1970 is 0.405805; 1971 is 0.728516. Because I_1964_ > I_1965_ > I_1966_ and I_1967_-I_1966_ > 0.1, 1968 is in the outbreak state. Similarly, I_1968_ > I_1969_ > I_1970_ and I_1971_-I_1970_ > 0.1, so 1972 is in the outbreak state.

**Fig. 3:**
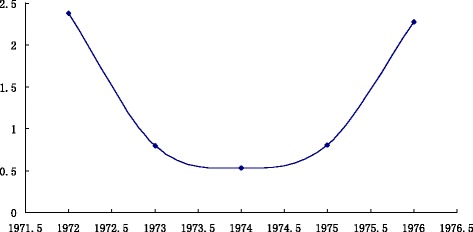
*Trend Chart* of composite index values from 1972 ~ 1976. *Horizontal axis* represents the year from 1972 ~ 1976, *vertical axis* represents the composite index value

The influenza A broke out in The United States, Russia and Japan in 1976 and 1977. Although the prevalence of this flu was typical of the outbreak, adults were slightly infected, and the incidence rate was very high in young people. As shown in Fig. 3, in 1972, the composite index value is 2.379322; 1973 is 0.79888; 1974 is 0.527835; 1975 is 0.801294. I_1972_ > I_1973_ > I_1974_ and I_1975_-I_1974_ > 0.1, so 1976 is in the outbreak state.

**Fig. 4:**
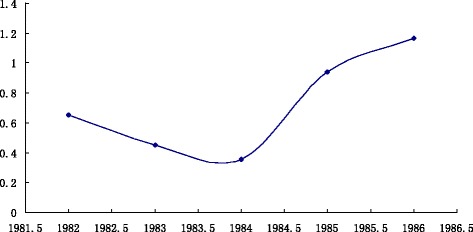
*Trend Chart* of composite index values from 1982 ~ 1986. *Horizontal axis* represents the year from 1982 ~ 1986, *vertical axis* represents the composite index value

The influenza A broke out in The United States and Japan in 1986. Meanwhile, many countries in Asia and Europe had the outbreak of influenza A. As shown in Fig. 4, in 1982, the composite index value is 0.650454; 1983 is 0.449789; 1984 is 0.354632; 1985 is 0.939702. I_1982_ > I_1983_ > I_1984_ and I_1985_-I_1984_ > 0.1, so 1986 is in the outbreak state.

**Fig. 5:**
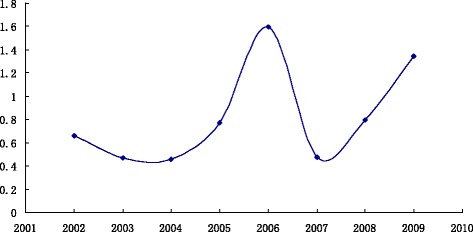
Trend Chart of composite index values from 2002 ~ 2009. *Horizontal axis* represents the year from 2002 ~ 2009, vertical axis represents the composite index value

The influenza A broke out in China in 2006. Global influenza pandemic caused by the new influenza A virus in 2009, of which 0.3 million people died [13, 14]. As shown in Fig. 5, in 2002, the composite index value is 0.660193; 2003 is 0.465805; 2004 is 0.45421; 2005 is 0.772595; 2006 is 1.595902; 2007 is 0.476057; 2008 is 0.798138. I_2002_ > I_2003_ > I_2004_ and I_2005_-I_2004_ > 0.1, I_2006_ > I_2007_ and I_2008_-I_2007_ > 0.1, so 2006 is in the outbreak state. Although I_2005_ is not larger than I_2006_, 2006 is outbreak year and other conditions are in line, so there is still the outbreak state in 2009.

**Fig. 6:**
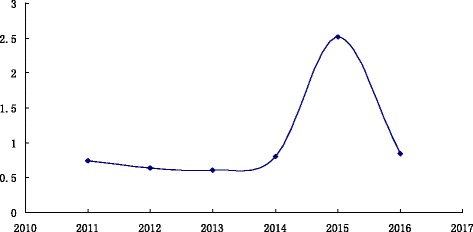
Trend Chart of composite index values from 2011 ~ 2016. Horizontal axis represents the year from 2011 ~ 2016, vertical axis represents the composite index value

The influenza A broke out in India in 2015, of which 1.5 thousand people died [15]. As shown in Fig. 6, in 2011, the composite index value is 0.740067; 2012 is 0.63573; 2013 is 0.6060092; 2014 is 0.806321. I_2011_ > I_2012_ > I_2013_ and I_2014_-I_2013_ > 0.1, so 2015 is in the outbreak state.

In general, the composite index varies along with the state of pandemic influenza virus from a relatively steady state to critical state before outbreak and then to the outbreak state. When the composite index continuous decreases for 2 years and increases of more than o.1 suddenly, it means the next year is normally in the outbreak state. Therefore, we can predict and identify whether a certain year is in the critical state before influenza A outbreak or outbreak state by observing the variation of index value.

### Forecasting influenza A pandemic outbreak in pandemic occurrence place

Take China as an example. The flu broke out in Hong Kong in 1968 and continued until 1969, of which 7.5 million people died. In 1972, influenza broke out in Henan Province and quickly spread to the entire province. As shown in Table 2, the data in 1965 and 1967 are absent, so we cannot forecast. 1968 is 2.198217; 1969 is 1.217563; 1970 is 0.645643; 1971 is 0.756451. I_1968_ > I_1969_ > I_1970_ and I_1971_-I_1970_ > 0.1, so 1972 is in the outbreak state.

Many countries in Asia including China had the outbreak of influenza A in 1986. As shown in Table 2, 1982 is 0.566947; 1983 is 0.489632; 1984 is 0.467346; 1985 is 0.896537. I_1982_ > I_1983_ > I_1984_ and I_1985_-I_1984_ > 0.1, so 1986 is in the outbreak state.

The influenza A broke out in China in 2006 and 2009. As shown in Table 2, 2002 is 0.673956; 2003 is 0.653958; 2004 is 0.543824; 2005 is 0.854835; 2006 is 1.632657; 2007 is 0.549367; 2008 is 0.924375. I_2002_ > I_2003_ > I_2004_ and I_2005_-I_2004_ > 0.1, I_2006_ > I_2007_ and I_2008_-I_2007_ > 0.1, so 2006 and 2009 are in the outbreak state.

## Conclusions

We select the data of protein amino acid sequence of pandemic influenza virus between 1934 and September 2016, and the different countries’ data such as China’s data between 1957 and 2015 in which only some data in a very few years are absent, and obtain a composite index by using PDNB. Although the amino acid sequence of each protein will randomly fluctuate, the composite index can still provide reliable, significant early warning information when influenza pandemic is close to the critical state or outbreak state. The network markers and other traditional markers cannot provide an early warning signal of the critical state before pandemic outbreak in comparison with dynamic network biomarker. This fully shows the dynamic network biomarker is more stable and accurate to determine the state in which the pandemic influenza virus, particularly the critical state of pandemic influenza. This will achieve the aim of early warning and then strengthen preventive measures in advance. This is of great significance for the research and warning of pandemic influenza virus.

## Abbreviations

DNB: Dynamical network biomarkers
PDNB: Protein dynamical network biomarkers
